# Comparative Effectiveness of Pitavastatin Versus Atorvastatin on Lipid Profile and Blood Sugar in Patients of Diabetic Dyslipidemia: An Open-Label Comparative Study

**DOI:** 10.7759/cureus.90307

**Published:** 2025-08-17

**Authors:** Gayatri Devi, Jatinder Singh, Baljinder Pal S Bal, Shilpa Chaudhary

**Affiliations:** 1 Pharmacology and Therapeutics, Employees' State Insurance Corporation (ESIC) Medical College and Hospital, Faridabad, IND; 2 Pharmacology and Therapeutics, Government Medical College, Amritsar, Amritsar, IND; 3 Internal Medicine, Government Medical College, Amritsar, Amritsar, IND; 4 Pharmacology and Pharmacovigilance, Employees' State Insurance Corporation (ESIC) Medical College and Hospital, Faridabad, IND; 5 Pharmaceutical Sciences, Delhi Pharmaceutical Sciences and Research University, New Delhi, IND

**Keywords:** cost effectiveness, diabetic dyslipidemia, efficacy, glycemic control, lipid profile, pitavastatin

## Abstract

Introduction*: *Diabetes, a chronic metabolic disorder, is one of the fastest-growing health emergencies. Dyslipidemia associated with diabetes increases the risk of hypertension, kidney disease, and cardiovascular diseases. So, dyslipidemia should be treated aggressively as a part of diabetic care. This study was framed to assess the comparative effectiveness of pitavastatin versus atorvastatin on lipid profiles and glycemic control in patients with type 2 diabetes mellitus (T2DM) and dyslipidemia, as well as their comparative cost-effectiveness.

Methods*: *This prospective, parallel, open-label, randomized study was conducted at Guru Nanak Dev Hospital Amritsar, India, between September 2020 to March 2022, which enrolled 84, type 2 diabetes mellitus (T2DM) patients aged 35-70 years with glycated hemoglobin (HbA1c) ≥6.5%, who were already taking a combination of metformin and glimepiride at a daily dosage of 1500-3000 mg and 1-2 mg respectively, for ≥3 months. Patients were randomly recruited at a 1:1 ratio in two groups, A (n=40) and B (n=44), to receive either pitavastatin (4 mg, group A) or atorvastatin (20 mg, group B) for 12 weeks. During the course of the study, 10 patients from group A and 14 patients from group B were excluded. The primary endpoint was a change in lipid profile levels, whereas the secondary endpoints were changes in blood glucose levels and adverse drug reactions.

Results: Increment of high-density lipoprotein (HDL) levels in pitavastatin (group A) was significantly more pronounced as compared to the atorvastatin (group B) (16.2 ± 5.0 versus 9.2 ± 4.5, p-value<0.0001), while in decrement of total cholesterol (TC) (17.5 ± 8.9 versus 20.1 ± 6.8%, p-value=0.211), low density lipoprotein (LDL) (25.0 ± 11.3 versus 29.1 ± 9.8%, p-value=0.140), and triglycerides (TGs) (20.8 ± 7.2 versus 18.5 ± 9.4%, p-value=0.281), there was no significant difference between two groups after 12 weeks of study. Regarding blood glucose levels, pitavastatin (group A) causes significant improvement in fasting blood glucose than atorvastatin group (22.7 ± 11.6 versus 15.4 ± 6.4%, p-value=0.004) and also the percent change in HbA1c from zero to 12 weeks was more in pitavastatin group than in atorvastatin group (6.4 ± 1.9 versus 5.4 ± 2.3, p-value=0.09). The cost-effectiveness of pitavastatin and atorvastatin was also compared, taking into account the percentage improvement of each parameter.

Conclusion: Pitavastatin was found to be more cost-effective than atorvastatin in reducing fasting blood glucose, HbA1c, and triglyceride levels, and in increasing HDL-cholesterol levels.

## Introduction

Diabetes is a chronic metabolic disorder characterized by persistent hyperglycemia, which may result in severe complications like heart disease, renal failure, and vision loss. It is currently one of the fastest-growing health emergencies globally and poses a significant public health concern in India. The number of individuals with diabetes in India is projected to rise from 74.2 million in 2021 to 124.9 million by 2045 [1].

The Indian population is more susceptible to developing diabetes due to factors such as genetic predisposition, rapid urbanization, increased abdominal obesity due to physical inactivity, and a higher prevalence of metabolic risk factors like hypertension and dyslipidemia [2].

Dyslipidemia related to diabetes significantly increases the chances of hypertension, metabolic syndrome, kidney disease, and cardiovascular disease (CVD) risks. In individuals with diabetes, a linear relationship exists between cholesterol levels and CVD, regardless of the baseline cholesterol levels; therefore, reducing the low-density lipoprotein (LDL) will reduce the risk of heart disease. Hence, dyslipidemia should be treated aggressively as a part of diabetic care. The American Diabetes Association recommends adding statins, regardless of LDL level, to patients over 40 years old with cardiovascular risk factors [3]. Epidemiological studies have shown that high-density lipoprotein (HDL)-cholesterol levels are inversely correlated with the risk of cardiovascular events [4].

Within the lipid profile, the primary target is to lower LDL-cholesterol, initially through lifestyle interventions and subsequently with pharmacological therapy. Statin therapy is the recommended first-line pharmacological treatment [5]. Statins (atorvastatin, simvastatin, rosuvastatin, pravastatin, etc.) act by inhibiting 3-hydroxy-3-methylglutaryl coenzyme A (HMG Co-A) reductase.

Atorvastatin, a potent LDL-lowering agent, is primarily used for the management of hypercholesterolemia associated with type 2 diabetes mellitus. Atorvastatin is one of the most widely prescribed drugs and the most widely prescribed statin in the world [6].

Statins can compromise glycemic control by decreasing levels of metabolites, such as isoprenoids, which are known to enhance glucose uptake by upregulating SLC2A4 expression. This gene encodes the membrane transporter protein glucose transporter 4 (GLUT4). This protein plays a role in glucose uptake by adipocytes. Recently, atorvastatin has been shown to inhibit adipocyte maturation and SLC2A4, which in turn inhibit GLUT4 expression and can potentially impair glucose tolerance [7].

Atorvastatin therapy at higher doses and for longer duration was found to cause glucose intolerance in normoglycemic persons and caused progression towards diabetes in prediabetic individuals [8].

So, in diabetic dyslipidemia, the ideal statin should possess properties that allow for both a reduction in total cholesterol and low-density lipoprotein cholesterol (LDL-C), as well as an increase in high-density lipoprotein cholesterol (HDL-C), a favorable effect on glycemic control, a low potential for drug-drug interactions, fewer adverse effects, and cost-effective.

Pitavastatin, a novel statin with a cyclopropyl moiety, is distinct from other statins. This moiety accounts for the potency of the molecule, appears to protect it from metabolism by CYP3A4, provides optimal activity as an HMG-Co-A reductase inhibitor, and facilitates better drug absorption, minimal drug-drug interactions, and a low incidence of adverse effects. It also improves endothelial function, increases nitric oxide production, inhibits smooth muscle cell contraction, and promotes apolipoprotein A-1 production, with a potent LDL-cholesterol reduction effect, sustained HDL-C raising, and antioxidant effects. The cyclopropyl moiety in pitavastatin enhances insulin resistance, promotes adiponectin release, and improves glucose metabolism and insulin sensitivity. Additionally, it helps maintain the proper development and function of adipocytes and plays a role in glucose uptake. Pitavastatin could provide an alternative treatment choice, especially in diabetic dyslipidemia [9,10].

Findings of the LIVES Study show that pitavastatin is associated not only with a significant reduction in LDL-cholesterol but also consistently produces a marked and sustained increase in HDL-C concentration on long-term use, as well as improvements in glycated hemoglobin (HbA1c) and beneficial effects on cardio-renal functions [11].

Therefore, it is crucial to understand the effects of pitavastatin and atorvastatin on blood glucose and lipid profiles, as well as their side effects, given that only a few studies have been conducted to evaluate the safety and efficacy of pitavastatin versus atorvastatin in patients with diabetic dyslipidaemia.

The present study aims to conduct a comparative evaluation of the effects of pitavastatin (4 mg) versus atorvastatin (20 mg) to determine which statin is more effective, economical, and safer for patients with dyslipidemia and type 2 diabetes mellitus.

## Materials and methods

This prospective, open-label, block-randomized, interventional, and comparative study was conducted at the medicine outpatient department (OPD) of Guru Nanak Dev Hospital of Government Medical College, Amritsar. Approval for the research protocol was secured from the Institutional Ethics Committee of Government Medical College, Amritsar (GMC/IEC/279), dated August 18, 2020. The patients were selected for the study after obtaining their written informed consent. Confidentiality of the patient's information was maintained throughout the study. Patients enrolled in the study began in September 2020 and ended in March 2022. The inclusion criteria were: (1) patients diagnosed type 2 DM with (i) HbA1c ≥ 6.5%; (ii) fasting blood glucose (FBG) ≥110 mg/dl; (iii) triglycerides ≥150 mg/dl; (iv) high-density lipoprotein (men ≤40 mg/dl, women ≤50 mg/dl); (v) body mass index >30 kg/m^2^; (vi) those taking a combination of metformin (1 g to 3 g) and glimepiride (1 mg to 3 mg) for ≥3 months; (vii) those aged between 35 and 70 years of both genders; and (viii) those who have given informed consent in writing to participate in the study.

The exclusion criteria were: (1) those patients with type l diabetes mellitus and end-stage complications with diabetes mellitus; (2) hypothyroidism, nephrotic syndrome, gout, pancreatitis, uncontrolled hypertension (DBP >95 mmHg); (3) hypersensitivity to atorvastatin or pitavastatin; (4) those taking any other glucose-lowering agents than the combination of metformin and glimepiride; (5) history of muscle pain (fibromyalgia with associated raised creatine phosphokinase (CPK) levels); (6) pregnant and lactating women; (7) patients on oral contraceptive pills (OCP'S)/oestrogens, hormone replacement therapy; (8) patient with abnormal liver function and renal function tests; (9) concurrent use of inhibitors of CYP3A4 isoenzyme, i.e., antifungals, macrolides, calcium-channel blockers (dihydropyridines, i.e., nifedipine, amlodipine), cyclosporine, gemfibrozil, and grapefruit juice; (10) concurrent use of enzyme inducers-phenobarbitone, rifampicin, phenytoin, etc., and (11) those who did not give consent for the study.

Patients who met the eligibility criteria were randomly (block randomization) assigned to two groups, A and B, at a 1:1 ratio, with group A (n=40) and group B (n=44) patients. A computer-based dynamic allocation method was employed to ensure an equal distribution of baseline features, including age, gender, fasting blood glucose (FBG), HbA1c levels, and lipid profile. Group A received pitavastatin 4 mg once daily, while group B patients received atorvastatin 20 mg once daily. These doses were selected based on standard therapeutic regimens and on the basis of published clinical evidence for treating dyslipidemia in type 2 diabetes mellitus patients. Each tablet was taken orally once daily in the evening with a standardized 1/2 glass of water. Both groups were taking a combination of antidiabetic drugs, i.e., metformin (1 g to 3 g) plus glimepiride (1 mg to 3 mg), along with dietary modifications and physical activity for 12 weeks. A general piece of advice regarding dietary modification and half an hour of yoga was given to both group participants at the start of the study, additionally, all participants were also instructed to seek dietary advice from a dietician. After starting pitavastatin and atorvastatin, no changes were made to the doses of antidiabetic drugs to evaluate the precise results. Patients were advised to report immediately if they developed any symptoms related to drug side effects.

During patient recruitment, medical history and general and systemic examinations were conducted to identify any potential complications associated with diabetic dyslipidemia. Additionally, the patients underwent various essential investigations, including renal function tests, liver function tests, fasting blood glucose, HbA1c, lipid profile, electrocardiogram, and fundus examination. After obtaining initial baseline values at the start (zero weeks), patients were scheduled for a follow-up visit 12 weeks later. At this visit, they underwent similar examinations and investigations as those performed at the recruitment visit. Fasting blood glucose (FBG) was monitored every two weeks. Patients were advised to come to the hospital in the morning after fasting overnight for 12 hours. All relevant details were meticulously recorded in a pre-designed clinical proforma.

The investigations were conducted in the clinical laboratory of Guru Nanak Dev Hospital and were assessed in the Department of Pharmacology, Amritsar. Throughout the study, patients' well-being was monitored for any adverse drug events via mobile communication and regular in-person visits to the medicine OPD, where they received their treatment drugs. Any adverse effects that occurred were noted down. Additionally, the researchers maintained regular contact with the patients to ensure their well-being and adherence to the treatment and instructions.

The collected data were transformed into variables, coded, and entered into Microsoft Excel 2019 (Microsoft, Redmond, Washington, USA). The data were later analyzed and subjected to statistical examination with the Statistical Package for the Social Sciences (SPSS), Version 25 (IBM, Armonk, New York, USA). Quantitative data were presented as means along with their standard deviations, and the differences between the two groups were assessed using an unpaired Student's t-test. Qualitative information was represented in terms of frequency and percentage.

## Results

**Figure 1:**
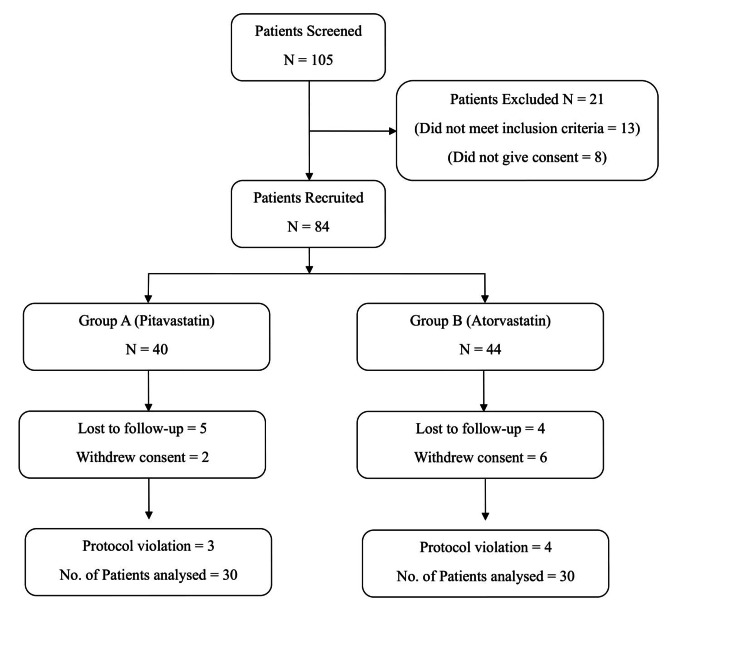
Flowchart showing patient enrollment, allocation, and analysis.

A total of 103 patients were screened for the study. Out of these, 21 were excluded-13 for not meeting the eligibility criteria and eight for not providing consent, leaving 84 patients who were eligible to participate. These eligible patients were randomly assigned to two groups: group A received pitavastatin, while group B received atorvastatin. In group A, there were 40 patients, and in group B, there were 44 patients. Ultimately, 30 patients from each group completed the treatment and were included in the final analysis (Figure 1).

During the course of the study, 10 patients from group A (five did not follow-up, two withdrew consent, and three violated protocol) and 14 patients from group B (four did not follow-up, six withdrew consent, and four violated protocol) were excluded. The baseline and demographic characteristics between the two study groups were comparable (Tables 1, 2).

**Table 1:** Baseline characteristics in study groups (mean ± SD) (n=60). SD: standard deviation, FBS: fasting blood glucose, HbA1c: glycated hemoglobin, TC: total cholesterol, TG: triglyceride, LDL: low-density lipoprotein, HDL: high-density lipoprotein.

Characteristics	Group A (Pitavastatin)	Group B (Atrovastatin)
Number of patients	30	30
Mean age (years)	56.2 ± 7.0	58.8 ± 6.4
Sex (M:F)	9:21	12:18
FBG (mg/dl)	172.9 ± 49.3	173.0 ± 39.3
HbA1c (%)	7.34 ± 0.53	7.22 ± 0.34
TC (mg/dl)	227.2 ± 35.1	215.5 ± 26.5
TG (mg/dl)	210.4 ± 45.1	198.5 ± 23.6
LDL-C (mg/dl)	146.9 ± 32.7	136.3 ± 26.1
HDL-C (mg/dl)	38.0 ± 4.4	39.5 ± 4.2

**Table 2:** Demographic characteristics of sample population (mean ± SD). df: degree of freedom. *Using two-sample t-test. **Using chi-square test.

Characteristics	Group A (pitavastatin)	Group B (atorvastatin)	All patients combined	Test statistic (t-value, df)	p-value
Mean age (years)	56.2 ± 7.0	58.8 ± 6.4	57.6 ± 6.8	t=-1.57, df=58	0.121*
Sex (M:F)	9:21	12:18	21:39	χ²=0.66, df=1	0.417**

Regarding lipid parameters, decrement in total cholesterol (17.5 ± 8.9 versus 20.1 ± 6.8%, p-value=0.211), low-density lipoprotein (25.0 ± 11.3 versus 29.1 ± 9.8%, p-value=0.140), and triglycerides (20.8 ± 7.2 versus 18.5 ± 9.4%, p-value=0.281). Statistically, there was no significant difference between the two groups after 12 weeks of treatment. The increment of high-density lipoprotein levels in pitavastatin (group A) was significantly more pronounced than that of atorvastatin (group B) (16.2 ± 5.0 versus 9.2 ± 4.5, p-value<0.0001) (Table 3).

**Table 3:** Comparison of mean change in lipid parameters from baseline to 12 weeks in two treatment groups. SD: standard deviation, FBS: fasting blood glucose, HbA1c: glycated hemoglobin, TC: total cholesterol, TG: triglyceride, LDL: low-density lipoprotein, HDL: high-density lipoprotein, df: degree of freedom. *Using two-sample t-test.

Lipid parameters (mg/dl)	Pitavastatin (group A) n=30			Atorvastatin (group B) n=30			Statistic values	
	Baseline (0 week) Mean ± SD	12 weeks Mean ± SD	Mean% change (0-12 weeks)	Baseline (0 week) Mean ± SD	12 weeks Mean ± SD	Mean % change (0-12 weeks)	Test statistic (t-value, df)	*p-value
TC	227.2 ± 35.1	185.4 ± 20.3	17.5 ± 8.9	215.5 ± 26.5	171.5 ± 19.1	20.1 ± 6.8	t=-1.25, df=58	0.211
LDL-C	147.1 ± 32.3	108.3 ± 19.2	25.0 ± 11.3	136.3 ± 26.1	96.3 ± 20.0	29.1 ± 9.8	t=-1.52, df=58	0.140
TG	210.4 ± 45.1	165.4 ± 31.8	20.8 ± 7.2	198.5 ± 23.6	160.7 ± 17.5	18.5 ± 9.4	t=0.57, df=58	0.281
HDL-C	38.0 ± 4.3	44.1 ± 4.4	16.2 ± 5.0	39.5 ± 4.2	43.0 ± 3.8	9.2 ± 4.5	t=4.31, df=58	<0.0001

Regarding blood glucose levels, in the pitavastatin group (group A), there was a substantial improvement in fasting blood glucose compared to the atorvastatin group (22.7± 11.6 versus 15.4 ± 6.4%, p-value=0.004). Also, the per cent change in HbA1c from zero to 12 weeks was greater in the pitavastatin group than in the atorvastatin group (6.4 ± 1.9 versus 5.4 ± 2.3, p-value =0.094). A longer duration study is needed to make any conclusion (Tables 4, 5).

**Table 4:** Change in mean FBG every two weeks in two treatment groups. FBG: fasting blood glucose, df: degree of freedom. *Two-sample t-test.

Time period (weeks)	Pitavastatin (group A) Mean ± SD	Atorvastatin (group B) Mean ± SD	Test statistic (t-value, df)	*p-value
0	172.9 ± 49.3	173.0 ± 39.3	t=-0.014, df=58	0.988
2	164.9 ± 45.2	166.2 ± 33.7	t=-0.132, df=58	0.895
4	160.7 ± 44.8	162.9 ± 33.4	t=-0.212, df=58	0.833
6	152.9 ± 44.6	158.9 ± 32.1	t=-0.495, df=58	0.623
8	147.5 ± 41.0	153.5 ± 29.6	t=-0.941, df=58	0.519
10	139.3 ± 36.2	150.0 ± 29.3	t=-1.257, df=58	0.214
12	131.1 ± 35.8	144.9 ± 27.6	t=-1.662, df=58	0.102

**Table 5:** Comparison of mean change in blood glucose parameters from baseline to 12 weeks in two treatment groups. SD: standard deviation, FBS: fasting blood glucose, HbA1c: glycated hemoglobin, df: degree of freedom. *Using two-sample t-test.

Blood glucose parameters	Pitavastatin (group A) n=30			Atorvastatin (group B) n=30			Statistic values	
	Baseline (0 week) Mean ± SD	12 weeks Mean ± SD	Mean% change (0-12 weeks)	Baseline (0 week) Mean ± SD	12 weeks Mean ± SD	Mean % change (0-12 weeks)	Test statistic (t-value, df)	*p-value
FBS (mg/dl)	172.9 ± 49.3	131.1 ± 35.8	22.7 ± 11.6	173.0 ± 39.3	144.9 ± 27.6	15.4 ± 6.4	t=2.96, df=58	0.004
HbA1c (%)	7.34 ± 0.53	6.87 ± 0.41	6.4 ± 1.9	7.22 ± 0.34	6.82 ± 0.22	5.4 ± 2.3	t=1.72, df=58	0.094

The cost-effectiveness of pitavastatin and atorvastatin was also compared; the average price of 10 tablets of pitavastatin (4 mg) is 130 rupees, and that of atorvastatin (20 mg) is 140 rupees for 10 tablets. Both drugs were of Zydus Cadila brand, taken from a local wholesale retailer in September 2020. The treatment cost for 12 weeks has been calculated (pitavastatin: 13 × 84 = 1092 rupees, atorvastatin: 14 × 84 = 1176 rupees). The percentage improvement in each parameter was considered and used in calculating the cost-effectiveness. Pitavastatin reduced fasting blood glucose, HbA1c, and triglycerides and increased HDL-c more cost-effectively than atorvastatin. On the other hand, atorvastatin is more cost-effective than pitavastatin in reducing total cholesterol and LDL-cholesterol (Table 6).

**Table 6:** Cost per percent improvement in various parameters in rupees (cost-effectiveness). FBS: fasting blood glucose, HbA1c: glycated hemoglobin, TC: total cholesterol, TG: triglyceride, LDL: low-density lipoprotein, HDL: high-density lipoprotein.

Parameter	Pitavastatin (group A)		Atorvastatin (group B)		Difference %	Cost-effective drug
	Mean percent improvement in parameter	Cost per percent improvement	Mean percent improvement in parameter	Cost per percent improvement		
FBG	22.7	48.11	15.4	76.36	58.7	Pitavastatin
HbA1c	6.4	170.63	5.4	217.78	27.6	Pitavastatin
TG	20.8	52.50	18.5	63.57	21.1	Pitavastatin
TC	17.5	62.40	20.1	58.51	6.7	Atorvastatin
LDL-C	25.0	43.68	29.1	40.41	8.1	Atorvastatin
HDL-C	16.2	67.41	9.2	127.83	89.6	Pitavastatin

During the study, four out of 30 patients (13.3%) in the pitavastatin group and seven out of 30 patients (23.3%) in the atorvastatin group reported adverse events (AEs). The nature of AEs was mild, such as myalgia, weakness, dyspepsia, and nausea; these were self-limiting and didn't require additional treatment, and didn't alter the treatment protocol. No serious adverse events have been reported in either group (Table 7).

**Table 7:** Comparison of adverse events between the treatment groups.

Side effects	Pitavastatin (group A) (n=30)	Atorvastatin (group B) (n=30)
Myalgia	1	3
Weakness	2	2
Dyspepsia	1	1
Nausea	0	1
	4 (13.3%)	7 (23.3%)

## Discussion

This randomized, prospective, parallel, and open-label study aimed to evaluate the comparative effectiveness of pitavastatin versus atorvastatin on lipid profile and blood sugar levels in patients with diabetic dyslipidemia. This study enrolled 60 patients with type 2 diabetes mellitus with deranged lipid profiles. After 12 weeks of treatment, it was found that pitavastatin is more efficacious than atorvastatin in increasing HDL and glycemic control, in terms of lipid profile. However, TC and LDL-cholesterol levels were decreased more in the atorvastatin group (group A), and TGs in the pitavastatin group (group B). Still, statistically, there was no significant difference between the two groups. The cost-effectiveness of pitavastatin and atorvastatin was also compared; this study found that pitavastatin is more cost-effective than atorvastatin in managing patients with diabetic dyslipidemia. Pitavastatin reduced fasting blood glucose, HbA1c, and TGs and increased HDL-C more cost-effectively than atorvastatin. On the other hand, atorvastatin is more cost-effective than pitavastatin in reducing total cholesterol and LDL-cholesterol levels. During this study, both medicines were well tolerated by the patients, and no serious adverse events were observed during this period.

A randomized, open-label, prospective comparative study of the efficacy and safety of pitavastatin versus atorvastatin was conducted in 100 patients of dyslipidemia in the medicine department of a tertiary care teaching hospital in Gujarat, in which atorvastatin 10 mg was given once in a day and pitavastatin 2 mg was given once in a day for 12 weeks, pitavastatin was more efficacious than atorvastatin in increasing HDL-C levels (p=0.028, i.e., p<0.05), while there was no significant difference between two groups in decreasing TC (p=0.567), LDL-cholesterol (p=0.615) and TGs (p=0.502), also both drugs were well tolerated. Adverse drug reactions were mild and subsided without treatment [12]. This study supports our study, as the current study also found that pitavastatin (4 mg) is more efficacious than atorvastatin (20 mg) in increasing high-density lipoprotein levels (16.2 ± 5.0 versus 9.2 ± 4.5, p-value<0.0001). In contrast, in the case of TC (17.5 ± 8.9 versus 20.1 ± 6.8, p-value 0.211), LDL-cholesterol (25.0 ± 11.3 versus 29.1 ± 9.8%, p-value=0.140) and TGs (20.8 ± 7.2 versus 18.5 ± 9.4, p=0.281) there is no significant difference between the two groups, also in the current study, both drugs were well tolerated. No serious adverse drug reactions were reported in either group. The only difference between our study and this study is the dosage of the drugs.

Similarly, a PIAT study conducted by Sasaki et al. compared atorvastatin 10 mg versus pitavastatin 2 mg for 52 weeks. The percent change in HDL-C levels was significantly greater in the pitavastatin group compared with the atorvastatin group (8.2 versus 2.9, respectively; P=0.031), the percent change in LDL-C levels was significantly lower with atorvastatin compared with pitavastatin (−40.1 versus −33.0, respectively; p=0.002), there were no significant differences between treatments concerning the measures of glucose metabolism [13]. In the current study too, pitavastatin (4 mg) is also more efficacious than atorvastatin (20 mg) in increasing high-density lipoprotein levels HDL (16.2 ± 5.0 versus 9.2 ± 4.5, p-value<0.0001). However, LDL-cholesterol levels were decreased more in the atorvastatin group (25.0 ± 11.3 versus 29.1 ± 9.8%, p-value=0.140), but statistically, there was no significant difference between the two groups. In blood glucose levels, in pitavastatin (group A) there was substantial improvement in fasting blood glucose than atorvastatin group (22.7± 11.6 versus 15.4 ± 6.4%, p-value=0.004), also the percent change in HbA1c from zero to 12 weeks was more in pitavastatin group than in atorvastatin group (6.4 ± 1.9 versus 5.4 ± 2.3, p-value=0.094), these findings of current study are in contrast to the above study, doses used in our study were of double strength as compared to the above research. Both drugs were very well tolerated, and no major adverse drug reactions were reported in either group.

The CHIBA study was a multicenter, collaborative, randomized, parallel-group study comparing pitavastatin and atorvastatin in terms of efficacy and safety in Japanese patients with hypercholesterolemia. Japanese patients with total cholesterol (TC)> 220 mg/dL were randomized to receive pitavastatin 2 mg (n=126) or atorvastatin 10 mg (n=125) for 12 weeks. Both pitavastatin and atorvastatin had significantly reduced the levels of LDL-C by 42.6% and 44.1%, TC by 29.7% and 31.1%, and TG by 17.3% and 10.7%, respectively, at 12 weeks., also HDL-C showed a significant increase at 12 weeks with pitavastatin treatment (3.2%, P=0.033 versus baseline) but not with atorvastatin treatment (1.7%, P=0.221 versus baseline). Both statins were tolerated well [14]. The findings of this study are similar to our current study, as in our study, there was also no statistically significant difference between the two groups in lowering TC, TGs, and LDL-cholesterol. Pitavastatin (4 mg) is more efficacious than atorvastatin (20 mg) in increasing high-density lipoprotein levels HDL (16.2 ± 5.0 versus 9.2 ± 4.5, p-value<0.0001).

In a sub-analysis of the CHIBA study, it was found that in the atorvastatin group, serum glycoalbumin had significantly increased compared to baseline (p=0.026), and HbA1c levels also tended to increase (p=0.098) in the atorvastatin group, which is in contrast to our study, but in the pitavastatin group, no significant change was detected in glycoalbumin. Improvements were shown in glucose tolerance parameters [15]. 

In COMPACT-CAD a 30-month study, improvements in HDL-C levels was significantly greater in pitavastatin group as compared to atorvastatin group (% change: pitavastatin: 20.1 ± 25.7%, atorvastatin: 6.3 ± 19.8%, p=0.01; absolute change: pitavastatin: 7.3 ± 9.1mg/dl, atorvastatin: 2.3 ± 8.0mg/dl, p=0.02). These findings are similar to the findings of our study (pitavastatin 16.2 ± 5.0 versus atorvastatin 9.2 ± 4.5, p-value<0.0001). Also, the treatment with pitavastatin substantially increased adiponectin levels. Neither statin had a significant effect on hemoglobin A1c, but in the current study, pitavastatin had reduced HbA1c levels. No severe adverse drug reactions were registered during the study [16], as in our study.

A study in Europe, conducted in 821 patients, showed no significant difference between the two groups (atorvastatin and pitavastatin) in the percent change in HDL-C levels (4% and 3%, respectively; p=0.840) [17]. This finding contradicts our study.

The present study had numerous limitations. First, it was an open-label study; there was no blinding, no placebo control. Second, the sample size of this study was small, with a short duration of follow-up. Third, the sample size was not calculated; had the study involved a larger number of participants and been of a longer duration, it could have been more impactful to support practice changes. Fourth, strict individual diet and physical activity were not standardized.

## Conclusions

Pitavastatin is more cost-effective than atorvastatin in reducing fasting blood glucose, HbA1c, and triglyceride levels and in increasing HDL-cholesterol levels. On the other hand, atorvastatin is more cost-effective than pitavastatin in lowering total cholesterol and LDL cholesterol levels. The adverse events were fewer in the pitavastatin group, as compared to the atorvastatin group.
